# ABC-YOLO: Automated skin burn depth classification using YOLO architectures

**DOI:** 10.1371/journal.pone.0344042

**Published:** 2026-03-18

**Authors:** Uğur Şevik, Onur Mutlu

**Affiliations:** 1 Department of Computer Science, Faculty of Science, Karadeniz Technical University, Trabzon, Türkiye; 2 Retina R&D Software and Engineering Services Ltd., Trabzon Teknokent, Trabzon, Türkiye; Peking University, CHINA

## Abstract

Accurate classification of skin burn depth is vital for determining appropriate treatment and accelerating the healing process. This study conducts a comparative analysis of YOLO-based deep learning architectures for the automated classification of skin burns. Analyses were performed on a robust, multi-source dataset created by combining a proprietary collection of 358 retrospective images from Karadeniz Technical University Farabi Hospital with two large public datasets from Roboflow Universe and Kaggle. All images were meticulously labeled into four burn degrees by expert general surgeons. To enhance model performance and generalizability, various data augmentation and preprocessing techniques were applied. Segmentation-based versions of YOLOv8 and YOLOv11 with different architectural sizes (medium, large, extra-large) were evaluated using metrics such as precision, recall, F1-Score, and mAP. The findings revealed that the YOLOv11x-seg model demonstrated marked superiority over all other tested architectures, achieving an F1-Score of 0.87 and a mAP@0.5 of 0.91. Statistical analysis confirmed the significance of these results. The study demonstrates that the YOLOv11x-seg architecture offers significant potential as a rapid and objective decision support tool in clinical settings. This work makes an original contribution to improving burn diagnosis by integrating a state-of-the-art deep learning model into medical image analysis.

## Introduction

Burns are among the medical emergencies that cause various degrees of damage to the skin, which is the largest organ of the human body [1]. Such injuries not only cause physical damage but can also have psychological effects and negatively affect quality of life. Accurate classification of burn severity is vital for guiding the treatment process and providing appropriate medical intervention in a timely manner [2]. In traditional methods, burn severity is typically determined based on visual observations and experience [3]. However, these methods are often time-consuming, subjective, and error-prone. In addition, inaccurate burn assessments can lead to improper wound management and negatively affect the treatment process [4]. Misdiagnosis of burn depth can lead to delayed or unnecessary surgical intervention, which can complicate the patient’s recovery process [5]. Therefore, there is a growing need for automated systems that can quickly, accurately, and objectively assess burn severity [6].

**Fig 1:**
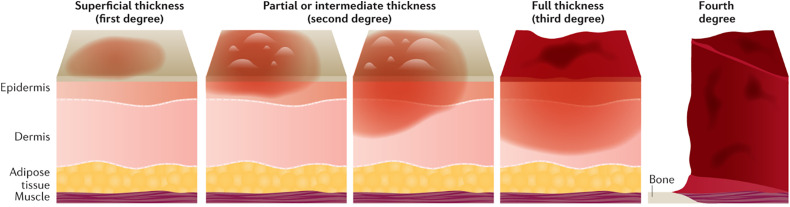
Visual classification of skin burn depth grades. Grade 1 (superficial), Grade 2 (superficial-partial or deep-partial thickness), Grade 3 (full thickness), and Grade 4 (deep tissue involvement, such as muscle or bone).

Burns have different depths and are generally classified into four main categories: 1st degree (superficial burns), 2nd degree (superficial-partial or deep-partial), 3rd degree (full thickness), and 4th degree (muscle and bone damage) burns [7,8], as illustrated in Fig 1 [9]. A 1st degree burn is the mildest type, in which only the skin surface, called the epidermis, is damaged and manifests itself with pain and redness [10]. 2nd degree burns affect the subcutaneous layer called the dermis, and fluid-filled blisters are formed [11]. 3rd degree burns can extend deep into the skin surface, causing tissue loss, and patients typically experience no pain because the nerve endings are destroyed [11]. 4th degree burns can progress to the muscles, bones, and other organs under the skin and are often life-threatening. Identifying these types of burns is crucial for treatment planning and monitoring of the healing process [12]. Therefore, accurate classification of burns ensures that appropriate treatment is administered and reduces the risk of complications. Methods such as laser Doppler imaging [13], harmonic ultrasound imaging [14], optical coherence tomography [15], and high-resolution infrared thermography [16] have been developed and incorporated into a limited clinical burn diagnosis to accurately assess burn depth. However, these technologies are generally not preferred in clinical settings due to their high costs and complexity. Instead, common cameras and smartphones can be used as practical alternatives for obtaining burn images.

In recent years, advanced artificial intelligence methods such as machine learning and deep learning have revolutionized the healthcare field [17]. By working on large datasets, these methods can exceed human capacity to solve complex problems. Particularly in the field of image analysis, deep learning algorithms allow the fast and accurate analysis of medical images [18]. Unlike traditional image processing techniques, deep learning algorithms can automatically extract meaningful features from images and perform object recognition and classification with high accuracy [19]. In this context, object detection algorithms play an important role in the automatic detection of burn severity [4].

The You Only Look Once (YOLO) series of algorithms is prominent in the field of object detection and segmentation, notable for their ability to provide high-speed, accurate, and computationally efficient visual data [20]. YOLOv8, an important version of this series, offers a balanced speed-accuracy performance and is preferred for achieving effective results on large-scale datasets. YOLOv11, on the other hand, offers high performance even in low-computational-power environments, which is especially advantageous in complex applications such as medical image analysis [21]. These algorithms detect objects in an image in a single step by processing the entire image simultaneously. This feature makes the YOLO series widely applicable in real-time applications. In particular, in cases such as burn severity detection, where accurate discrimination of color variations, texture differences, and depth variations in images is required, the combinational advantages (speed, accuracy, and efficiency) offered by the YOLO series of algorithms play a critical role.

Although existing studies utilizing CNNs and earlier YOLO versions have achieved promising results in burn classification, most rely solely on classification-based approaches that fail to precisely localize the burn area. Furthermore, there is a lack of research evaluating the efficacy of next-generation architectures, specifically YOLOv11, on complex medical datasets that combine diverse burn severities. Addressing this gap requires a segmentation-first approach that not only classifies burn depth but also delineates the lesion boundaries, thereby providing a more comprehensive tool for clinical decision support. This study bridges this gap by introducing a high-performance, segmentation-based ABC-YOLO framework trained on a robust multi-source dataset.

This study aimed to comparatively evaluate the usability of the YOLOv8 and YOLOv11 algorithms for automatic detection of burn severity. The wide compatibility and stable performance of YOLOv8 and the high efficiency of YOLOv11 with low resource consumption have the potential to provide practical solutions in clinical settings. Accurate classification of burn severity can contribute to the personalization of treatment protocols, accelerate recovery, and optimize the use of medical resources. By detailing the performance metrics (accuracy, precision, F1 score, and computation time) of the YOLO series models, this study aims to provide a guide on which model is more suitable for burn assessment in a clinical scenario [22]. In addition, suggestions were made for developing hybrid systems by integrating these models to guide future medical diagnostic technologies.

## Materials and methods

In this section, the skin-burns image dataset, the preprocessing techniques applied to it, the YOLO deep learning architectures used for burn-depth classification, the training and testing procedures for these architectures, and the performance metrics used to evaluate the results are presented in detail.

### Data set

To ensure the development of a robust and generalizable deep learning model and to address the limitations of a single-center dataset, this study utilized a large, combined, multi-source dataset. The dataset was constructed from three primary sources: a proprietary collection from a university hospital and two large, publicly available repositories (Roboflow Universe and Kaggle).

#### Data collection and sources.

The dataset used in this study was constructed from multiple sources to enhance robustness and generalizability. The primary component consists of proprietary skin burn images retrospectively obtained from the archives of the Department of General Surgery at Karadeniz Technical University Farabi Hospital, captured via standard digital photography during routine patient care between 2011 and 2015. For the purpose of this research, these images were accessed in November 2024. A formal waiver of ethical approval and individual patient consent was granted by the institutional review board (Protocol 2017/1) for the retrospective analysis of these images. To further increase data diversity and improve the model’s generalization capability, this proprietary collection was augmented with two publicly available skin burn datasets from Roboflow Universe and Kaggle. Prior to analysis, all images from all sources were fully anonymized to ensure compliance with patient privacy and data confidentiality principles, containing no identifying information such as names, protocol numbers, faces, or distinctive body markings.

**Fig 2:**
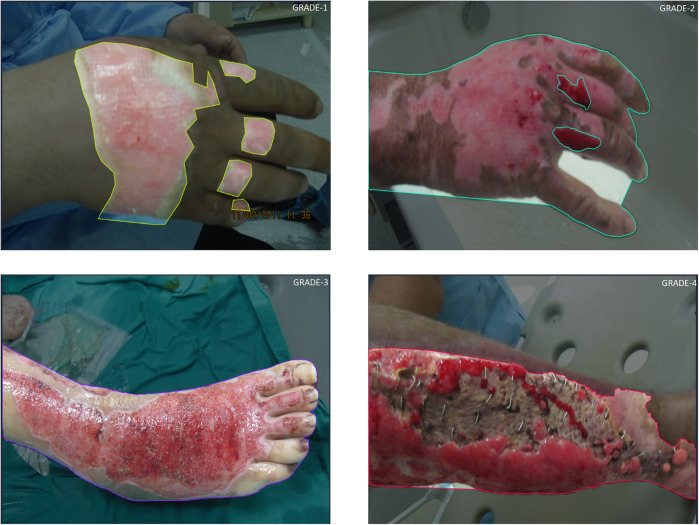
Some image examples were selected from the dataset used in the study, showing four different burn depth grades (Grade 1: Superficial; Grade 2: Superficial or Deep Partial Thickness; Grade 3: Full Thickness; Grade 4: Deep Tissue Involvement such as Muscle/Bone).

The image collection process encompassed a variety of cases with different degrees and appearances of burns. Fig 2 illustrates representative images selected from the dataset, showcasing all four burn degrees. Data privacy and patient confidentiality principles were strictly adhered to throughout the study.

This study was conducted in accordance with the principles of the Declaration of Helsinki. The study protocol was reviewed by the Karadeniz Technical University, Faculty of Medicine, Institutional Review Board. The board determined that this research, involving the retrospective analysis of fully anonymized and de-identified patient images, qualified for a formal waiver of ethical approval (Ethics approval: 2017/1, Clinical trial number: 24237859−26). As the data were fully anonymized and the analysis was retrospective, the ethics committee also waived the requirement for individual patient consent.

#### Labeling and quality control.

The labeling and quality control process was conducted in two stages to ensure a high-quality final dataset. Initially, the proprietary burn images from Karadeniz Technical University Farabi Hospital were independently classified by two board-certified general surgeons with burn expertise using a double-blind protocol. The surgeons performed the labeling with high intra-observer agreement, ensuring consistency in their individual assessments. The inter-observer agreement between the two experts for this local dataset was then assessed using the Cohen’s Kappa (κ) statistic, which yielded a value of κ = 0.94, indicating “almost perfect agreement” in the literature. In cases of disagreement (approximately 6% of the total images), the final label was determined by a third senior physician with expertise in the subject. Subsequently, the expert surgeons meticulously reviewed images from publicly available datasets. During this curation process, they excluded any images that were blurry, unclear, or where the burn degree was ambiguous and subject to debate. This rigorous two-stage labeling and quality-control process yielded a highly reliable final dataset for model training.

The label distribution by class and details of the balanced dataset generation strategy are presented in Table 1.

**Table 1:** Label distribution according to burn degree.

Classes	Proprietary Dataset (n)	Roboflow Dataset (n)	Kaggle Dataset (n)	Total (n)	Percentage (%)
**First-Degree**	83	157	106	346	23%
**Second-Degree**	119	219	196	534	36%
**Third-Degree**	105	259	164	528	36%
**Fourth-Degree**	51	19	0	70	5%
**Total (n)**	358	654	466	1478	100%

#### Data preprocessing.

To ensure optimal model performance and input compatibility, a comprehensive preprocessing pipeline was applied to the burn images. First, all raw images were resized to a standard 640 × 640 pixels to conform to the model’s input format and improve computational efficiency, using interpolation algorithms to preserve image integrity. Following resizing, pixel values were normalized to the (0, 1) interval to stabilize training and accelerate model convergence.

During the training phase, extensive data augmentation techniques were employed to mitigate the risk of overfitting and improve the model’s generalization. These techniques increase data diversity by generating synthetic samples from existing data. The primary methods applied included random horizontal flipping, random rotations within ±15°, and adjustments to image brightness and contrast. The data augmentation process was efficiently implemented using the Albumentations and PyTorch libraries.

#### Dataset partitioning.

A total of 358 labeled images were divided into 80% training (286 images) and 20% testing (72 images) using stratified random sampling to ensure a balanced representation of class distributions in the training and test sets. This approach was adopted to preserve the reliability of model evaluation, particularly for datasets with imbalanced classes. As a result of data augmentation, the training set was expanded to 1430 images, enabling the model to learn diverse patterns and mitigate the effects of data imbalance. The test dataset was not augmented to objectively assess the model’s generalization performance. The backbone–neck–head components of YOLOv8 are schematically presented in. 3.

### Segmentation architecture and model selection

For the purpose of automatic, accurate, and fast detection and classification of burn severity from burn images, two different advanced versions of the YOLO architecture, YOLOv8 and YOLOv11, which are up-to-date and high-performance in the fields of object detection and semantic segmentation, were chosen for comparative analysis. Both models were classified as one-stage detectors. This approach processes images in a single neural network pass, enabling simultaneous localization of regions of interest (via bounding boxes or segmentation masks) and estimation of their corresponding classes. This simultaneous, single-pass processing characteristic is a critical advantage, particularly for time-sensitive clinical applications, such as real-time decision support systems. Owing to their distinct architectural designs, varying computational complexities, and potential performance differences, these models are ideal candidates for a thorough evaluation of their effectiveness in sensitive and challenging medical imaging tasks, such as burn detection.

#### YOLOv8 architecture and technical specifications.

YOLOv8 represents an important step in the evolution of the YOLO (You Only Look Once) series. Owing to its flexibility, scalability, and various model sizes (nano ‘n’, small ‘s’, medium ‘m’, large ‘l’, extra-large ‘x’) that can be adapted to different computational infrastructures, YOLOv8 has gained wide acceptance in both academia and industry. The model exhibits high performance, particularly on large-scale datasets, owing to the optimized balance between speed and accuracy [23].

**Backbone**: This layer is based on an enhanced variant of the CSPDarknet53 architecture for feature extraction. Cross-stage partial (CSP) connections split the feature maps into two branches: one branch is passed directly to the next layer, whereas the other branch passes through a process of convolutional blocks. In addition to improving the efficiency of gradient flow, this strategy alleviates computational bottlenecks and increases the learning capacity of the model.

**Neck**: This section integrates the Path Aggregation Network (PANet) and Spatial Pyramid Pooling Fast (SPPF) modules to realize multi-scale feature fusion. The PANet module integrates low-level details and high-level semantic information in a bidirectional flow, whereas the SPPF module enriches the contextual information vocabulary through pooling layers applied at different scales.

**Head**: This section also serves as a prediction layer and employs an anchor-free, decoupled design. In this design, the classification, regression, and segmentation tasks are performed by separate processing arms, enabling more efficient optimization for each task. In addition, multi-scale object detection is performed by utilizing feature maps at different resolution levels (e.g., 80 × 80, 40 × 40, and 20 × 20 pixels).

**Optimization and Other Features**: During model training, AdamW is typically used as an optimization algorithm. The AdamW algorithm improves the model’s generalization performance by enhancing the effectiveness of standard L2 regularization in Adam optimization. For learning rate scheduling, a Cosine Annealing strategy (initial learning rate: 1 × 10^−3^, minimum learning rate: 1 × 10^−5^) was adopted. This strategy minimizes the probability that the model deviates from a local minimum by periodically and gradually decreasing the learning rate, thereby contributing to more stable convergence.

**Fig 3:**
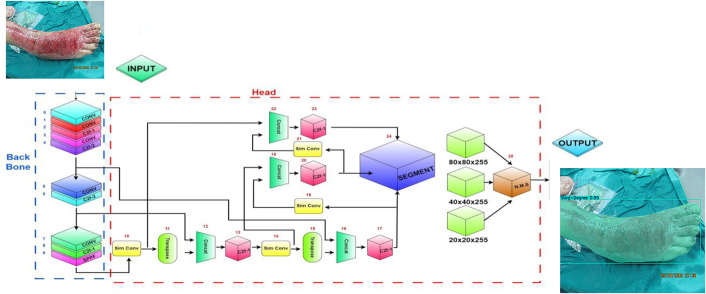
YOLOv8 based burn image segmentation architecture: Multi-scale feature extraction and generation of segmentation outputs through backbone, neck and head layers.

All these components, i.e., the backbone for feature extraction, the neck for feature fusion and enrichment, and the head for segmentation predictions, constitute the overall structure of the YOLOv8 architecture, which is schematically presented in Fig 3.

#### YOLOv11 architecture and specifications.

**Fig 4:**
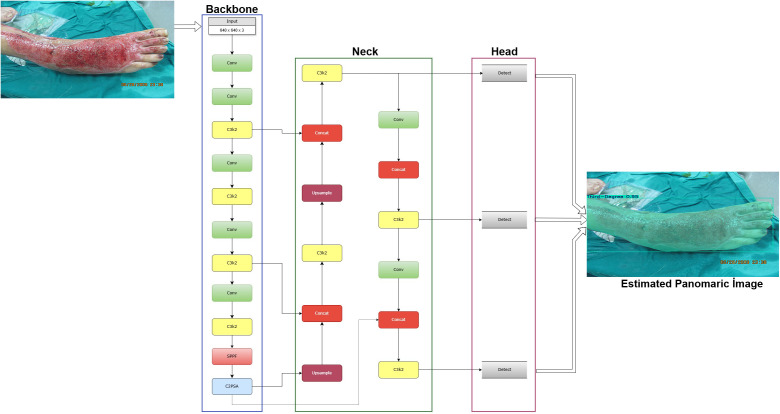
YOLOv11-based burn image segmentation architecture: Feature extraction, multilayer processing, and panoramic output estimation through backbone, neck, and head modules.

YOLOv11 is a next-generation object detection and segmentation model designed specifically with the goals of low resource consumption, high computational efficiency, and efficient performance on edge-computing devices [20]. This model was developed to operate efficiently without sacrificing performance, even in environments with limited processing capacity, such as mobile platforms, embedded systems, and standard clinical computers. The basic architectural structure and internal data flow diagram of YOLOv11 are shown in Fig 4.

**Backbone:** The backbone layer of the YOLOv11 architecture is based on the MobileNetv3-Large architecture, which is characterized by high computational efficiency. MobileNetV3 produces efficient and powerful feature representations through advanced mechanisms, including inverted residual blocks, linear bottlenecks, and Squeeze-and-Excitation (SE) modules. However, by replacing standard convolution operations with depth-wise separable convolutions, the number of parameters and computational complexity (in FLOPs) of the model are significantly reduced, whereas the quality of feature extraction is not compromised. This efficiency is based on the principle that depth-based convolution performs spatial filtering specific to each input channel, and pointwise convolution then combines these filtered feature maps.

**Neck:** In the neck layer of the model, a Bidirectional Feature Pyramid Network (BiFPN) architecture was adopted for the efficient and effective integration of multi-scale feature maps. BiFPN optimizes feature fusion by learnable weighting of features extracted at different resolution levels, enabling bidirectional information flow from both top-down and bottom-up approaches and yielding richer, more contextually robust feature maps. This is a significant advantage, particularly for high-precision burn detection in complex scenes with variable dimensions.

**Head:** The Dynamic Head module, located in the model’s output layer, employs attention mechanisms to provide adaptive focusing across both the channel and spatial dimensions. This enhances the model’s ability to detect difficult-to-detect objects, particularly in small, low-contrast, or partially occluded burn areas. The Dynamic Heading module can also dynamically modulate the relative importance of different feature levels.

**Model Optimization and Unique Techniques:** One of the key innovations of the YOLOv11 architecture is the advanced and efficient integration of channel pruning. This technique enables the automatic detection and elimination of filters that contribute little to the model’s overall performance or are redundant, either post-training or during training, resulting in a significant reduction in memory consumption and inference time. This optimization strategy significantly improves the model’s real-time inference performance on edge computing devices. During model training, in addition to the AdamW optimizer, a linear warm-up was employed to stabilize training by gradually increasing the learning rate in the initial phase. This combined approach enables a more stable initial phase and, consequently, higher final model performance, particularly when training models with complex architectures and large datasets.

Fig 4 shows the basic modules of the YOLOv11 architecture, such as the spine, neck, and head, as well as the feature extraction, multilayer processing, and panoramic output prediction processes performed using these modules.

### Performance measures and evaluation

Evaluating the performance of deep learning-based segmentation and classification models is not limited to basic classification metrics. In particular, problems such as object detection and segmentation, where both class labels and spatial information are estimated, require multidimensional evaluation approaches. Because such problems answer not only the “what” question but also the “where” question, more sophisticated metrics should be used in the evaluation process, including spatial accuracy, in addition to classical classification metrics.

In this context, the model’s performance on segmentation tasks is evaluated not only using basic metrics (accuracy, precision, and sensitivity) but also with advanced metrics (mean accuracy and mean average accuracy, mAP). This comprehensive analysis approach is of great importance for evaluating the validity and reliability of models under real field conditions, particularly in areas that require high accuracy, such as medical image processing.

One of the main tools for analyzing a model’s classification performance is the confusion matrix. This matrix compares the model’s predictions on the test data with the ground-truth class labels and shows the classes in which the model was correctly and incorrectly classified. The confusion matrix comprises four basic components.

**True Positive (TP):** The number of instances where the model correctly predicts positive (1) when the true class is positive (1). The model successfully recognizes a positive situation.

**TN (True Negative):** Number of instances in which the model correctly predicts negative (0) when the true class is negative (0). The model correctly recognized a negative situation.

**False Positive (FP):** The number of instances where the model incorrectly predicts positive (1) when the true class is negative (0). This is also referred to as a false alarm.

**False Negative (FN):** The number of instances where the model incorrectly predicts a negative (0), even though the true class is positive (1). This was considered a missed-positive case.

The main performance metrics calculated based on these values are as follows.

**Accuracy**: This indicates how many of the model’s predictions are correct.


$$\text{Accuracy }=\;\frac{TP+TN}{TP+FP+TN+FN}$$ (1)

**Recall**: Number of true-positive samples correctly predicted. This is particularly important in applications in which missed diagnoses are critical.


$$\text{Recall }=\;\frac{TP}{TP+FN}$$ (2)

**Precision**: Measures of how many of the samples predicted as positive were actually positive. False alarms were considered to be statistically significant.


$$\text{Precision }=\;\frac{TP}{TP+FP}$$ (3)

**F1-Score:** The F1 Score is the harmonic mean of Precision and Recall, offering a single metric that balances both. It is particularly valuable in imbalanced-class settings, as it accounts for both false positives and false negatives. The formula is as follows:


$$\text{F1}-\text{Score }=\;\text{2}*\;\frac{\text{Precision}\times \text{Recall}}{\text{Precision}+\text{Recall}}$$ (4)

#### Segmentation metrics: AP and mAP.

Because segmentation tasks require not only accurate object classification within an image but also precise localization, traditional classification metrics alone are inadequate for comprehensive evaluation. At this juncture, specialized metrics, such as Average Precision (AP) and Mean Average Precision (mAP), which can simultaneously measure both classification accuracy and positional precision, have become relevant.

**Average Precision (AP)** is a fundamental metric obtained by calculating the area under the precision-recall curve to evaluate a model’s performance for a specific class. This value encapsulates the model’s overall performance for the specified class in a single scalar, thereby facilitating comparative analysis. The mathematical expression for AP is as follows:


$$\text{AP }=\;\int_{\text{0}}^{\text{1}}\text{p}(\text{r})dr$$ (5)

where p(r) represents the precision value achieved by the model at a specific recall level, r.

Mean Average Precision (mAP) is a comprehensive performance indicator derived from multi-class object detection or segmentation problems by taking the arithmetic mean of Average Precision (AP) scores calculated for each specific class. Its variants, such as mAP@0.5 and mAP@[0.5:0.95], which are frequently used in the segmentation literature, provide a more comprehensive performance measure by evaluating the model’s performance across different Intersection over Union (IoU) thresholds.


$$\begin{array}{c} \text{mAP }=\;\frac{\text{1}}{N}\;\sum {\mathrm{\backslash nolimits}}_{i=\text{1}}^{N}AP_{i} \end{array}$$ (6)

where N denotes the total number of classes.

#### Calculation details.

**Fig 5:**
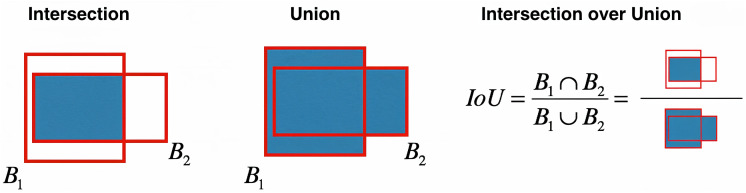
Calculation of the Intersection over Union metric [24].

**Intersection over Union (IoU):** As shown in Fig 5, it measures the overlap between the predicted segmentation mask and the actual mask.

mAP@0.5: Calculated mAP value when IoU threshold is 0.5.

mAP@0.5:0.95: Mean mAP obtained by a stepwise increase in the IoU threshold from 0.5 to 0.95.

These metrics are critical for assessing the reliability of the YOLO models in clinical scenarios. In particular, high mAP values demonstrated the model’s consistency across both classification and localization tasks. Furthermore, confusion matrix analysis enables the identification of classes in which the model is weak and the development of strategies for improvement.

### Computational infrastructure

The training and validation of the deep learning models used in this study were conducted on a system with high computational capacity and up-to-date hardware and software infrastructure. The system’s architectural details are presented comprehensively in this section to ensure reproducibility of the research findings and to provide transparency in the experimental process.

The core of this computational infrastructure consists of an NVIDIA GeForce RTX 4090 graphics processing unit (GPU) with 16,384 CUDA cores and 24 GB of GDDR6X video memory (VRAM), designed to meet the intensive parallel processing demands of deep learning models. A high VRAM capacity has significantly contributed to the optimization of training processes by enabling effective training of large-scale model architectures and large batches of high-resolution image data. The system’s overall processing performance was enhanced by an Intel Core i9-14900K central processing unit (CPU) with a 24-core, 32-thread architecture. Owing to its high clock speed, this CPU plays a central role in the efficient execution of data preprocessing, data loading, and other system tasks external to the GPU. This hardware configuration is completed with 64 GB of DDR5 RAM, which has successfully supported critical functions such as processing large-scale datasets, efficiently managing data communication between the CPU and GPU, and maintaining system performance stability during memory-intensive operations.

## Results

In this section, the efficacy of deep learning-based segmentation models, developed and comparatively analyzed within the scope of this study, in determining and classifying burn degrees are evaluated in light of comprehensive quantitative metrics and supporting visual analyses. The findings encompass the overall performance levels of the developed models and the detailed classification capabilities and learning dynamics of the top-performing model.

### Comparative model performances

The segmentation and classification performances of the YOLOv8-seg and YOLOv11-seg architectures developed specifically for this study, in their different scale configurations (medium ‘m’, large ‘l’, extra-large ‘x’), were compared based on standard evaluation metrics detailed in Table 2. These evaluation criteria encompass fundamental performance indicators such as precision, recall, overall accuracy, mAP@0.5, and mAP@0.5:0.95.

**Table 2:** Performance evaluation of YOLO-Seg based burn classification models.

Model	Precision(M)	Recall(M)	F1-Score(M)	mAP@0.5(M)	mAP@0.5:0.95(M)
**YOLOv8m-seg**	0.75	0.71	0.73	0.67	0.48
**YOLOv8l-seg**	0.79	0.76	0.77	0.76	0.59
**YOLOv8x-seg**	0.81	0.78	0.79	0.80	0.63
**YOLOv11m-seg**	0.64	0.71	0.67	0.71	0.49
**YOLOv11l-seg**	0.80	0.76	0.78	0.78	0.60
**YOLOv11x-seg**	**0.87**	**0.88**	**0.87**	**0.91**	**0.73**

Note: (M) Value at which the metrics were calculated using segmentation masks.

An analysis of the data in Table 2 indicates a general trend of performance improvement with increasing model size for both the YOLOv8 and YOLOv11 families.

Within the YOLOv8 architecture series, the YOLOv8m-seg model recorded a precision of 0.75, a recall of 0.71, an F1-Score of 0.73, an mAP@0.5 of 0.67, and an mAP@0.5:0.95 of 0.48. The larger YOLOv8l-seg model showed improved performance across all metrics, achieving a precision of 0.79 and mAP@0.5 of 0.76. The largest model in this family, YOLOv8x-seg, continued this trend, achieving a precision of 0.81, mAP@0.5 of 0.80, and mAP@0.5:0.95 of 0.63.

For the YOLOv11 architecture series, the YOLOv11m-seg model had a precision of 0.64 and a mAP@0.5 of 0.71. The YOLOv11l-seg model showed higher values, with a precision of 0.80 and mAP@0.5 of 0.78. The YOLOv11x-seg model produced the highest values across all tested models, with a precision of 0.87, recall of 0.88, F1-Score of 0.87, mAP@0.5 of 0.91, and mAP@0.5:0.95 of 0.73.

To rigorously evaluate the differences between the models, a statistical analysis was performed on their classification outputs. Given that all models were tested on the same dataset, paired statistical tests are appropriate for comparing their performance. Cochran’s Q test was employed to determine if a statistically significant difference existed among the six models simultaneously. This test compares the proportion of correct/incorrect classifications across all models and revealed a significant overall difference (p < 0.05), indicating that the models’ performances were not statistically identical. To further investigate pairwise differences, particularly between our top-performing model (YOLOv11x-seg) and the next-best model (YOLOv8x-seg), a post-hoc McNemar’s test was conducted. This test confirmed that YOLOv11x-seg made significantly fewer errors and was statistically superior to YOLOv8x-seg (p < 0.05). These analyses provide statistical evidence that the superior performance of the YOLOv11x-seg model is not attributable to chance and represents a significant improvement over other architectures.

**Fig 6:**
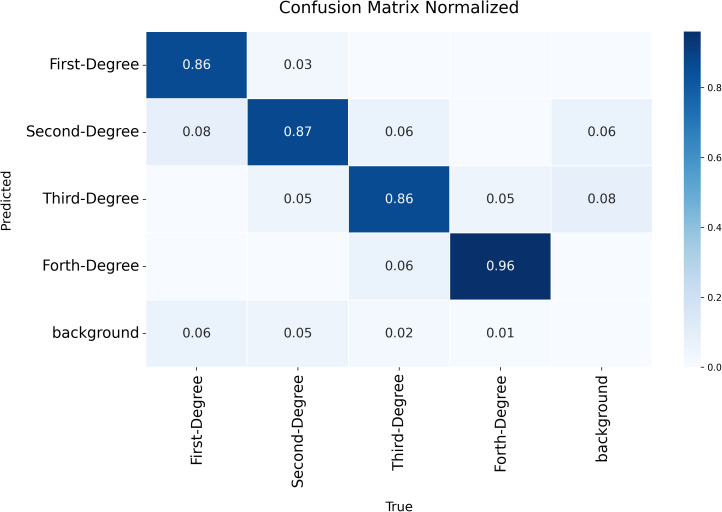
Confusion matrix for classification of burn degrees with YOLOv11x-seg.

The normalized confusion matrix in Fig 6 provides a detailed representation of the YOLOv11x-seg model’s ability to distinguish among the four burn degrees and the background class.

The values on the diagonal of the matrix show the rates at which the model correctly identified each burn degree. The model classified first-degree burns with 86% accuracy, second-degree burns with 87% accuracy, third-degree burns with 86% accuracy, and fourth-degree burns with 96% accuracy.

The matrix also details the misclassifications. For instance, 8% of true first-degree burns were predicted as second-degree. For second-degree burns, 3% were predicted as first-degree and 5% as third-degree. For third-degree burns, 6% were predicted as second-degree and 6% as fourth-degree. For fourth-degree burns, 5% were predicted as third-degree.

Additionally, the model classified some true burn regions as ‘background’. This occurred for 6% of first-degree, 5% of second-degree, 2% of third-degree, and 1% of fourth-degree burns.

**Fig 7:**
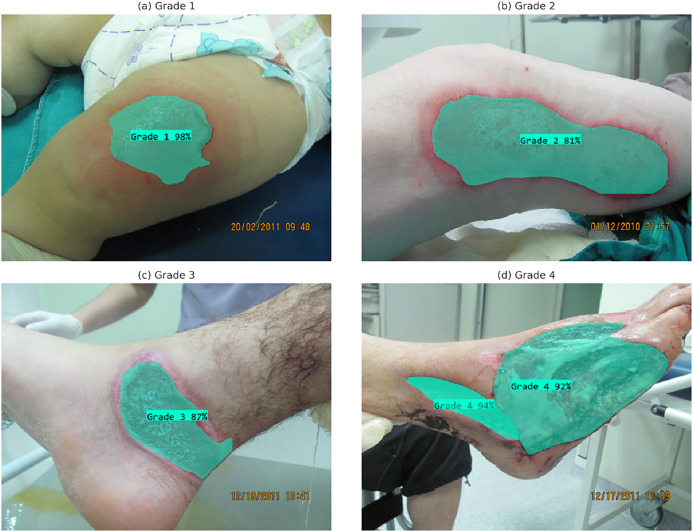
illustrates the representative segmentation outputs generated by the YOLOv11x-seg model for each of the four burn depth grades. These images, selected from the test dataset, visually demonstrate the model’s ability to localize and classify burn regions with high accuracy. In Fig 7a, the model correctly identified a superficial first-degree burn limited to the epidermis. Fig 7b shows a second-degree partial-thickness burn, in which dermal involvement and blistering are evident. In Fig 7c, the model successfully segments a third-degree burn, characterized by full-thickness skin loss and destruction of nerve endings. Fig 7d shows a fourth-degree burn extending into the underlying tissues, such as muscle and bone, with necrotic and grayish discoloration. The segmentation masks, overlaid in cyan, reveal the spatial extent and confidence level of the predictions, offering visual evidence of the model’s robustness and clinical applicability in distinguishing nuanced tissue-damage patterns.

Fig 7 Representative segmentation outputs for four different burn depth grades detected by YOLOv11x-seg model. (a) First-degree superficial burn. (b) Second-degree partial-thickness burn. (c) Third-degree full-thickness burn. (d) Fourth-degree burns involving deep tissues, such as muscle and bone.

## Discussion

This study comprehensively evaluated the performance of various variants of the current YOLO algorithms for the automatic detection and classification of burn depth in digital images. The empirical findings provide strong evidence for the practical applicability of the developed deep learning-based methodology in medical imaging. In particular, the superior performance of the YOLOv11x-seg model, achieved through optimization, has reaffirmed the significant potential of deep learning to address such complex medical challenges. With high success rates, including an mAP@0.5 of 0.91 and an F1-Score of 0.87, our model has the potential to provide a rapid, objective, and standardized alternative to traditional burn assessment methods, which currently rely on physicians’ individual experience and time-consuming visual examinations.

A closer analysis of the results reveals a distinct trend where model performance improved with increased architectural size, as evidenced by the progression from medium (‘m’) to extra-large (‘x’) variants in both YOLOv8 and YOLOv11 families. The superior performance of the YOLOv11x-seg model can be attributed to its advanced architecture, which incorporates an efficient MobileNetv3-Large backbone and a Bi-directional Feature Pyramid Network (BiFPN) neck, enhancing its feature extraction and fusion capabilities. However, despite its high overall accuracy, the model struggled to distinguish among adjacent burn degrees, which share visual similarities. The confusion matrix analysis showed that the model occasionally confused first- and second-degree burns, as well as third- and fourth-degree burns. This reflects the inherent difficulty of this fine-grained classification task. Furthermore, it is important to acknowledge that these results were obtained using a dataset captured under relatively consistent imaging conditions; model performance can be sensitive to variations in lighting, background, or imaging devices, which limits generalizability.

Our findings contribute to the “promising but developing frontier” of machine learning in burn wound evaluation, a field systematically reviewed by Huang et al. [4], who noted that most burn depth classification models achieve accuracies over 83%. To contextualize our results within the current landscape, **Table 3** presents a comprehensive comparison of our ABC-YOLO framework with recent state-of-the-art studies.

**Table 3:** Comparison of the proposed ABC-YOLO model with state-of-the-art studies.

Study	Year	Method	Task	Dataset Size	Performance
Karthik et al. [25]	2021	CNN-RNN	Classification	104	81.4% (Accuracy)
Suha & Sanam [2]	2022	VGG16	Classification	1,530	95.6% (Accuracy)
Boissin et al. [26]	2023	CNN	Classification	1,105	87.2% (Accuracy)
Yıldız et al. [27]	2024	YOLOv7	Segmentation	1,320	75.0% (mAP@0.5)
Proposed Method	**2025**	**YOLOv11x-seg**	**Segmentation**	**1,478**	**91.0%** (mAP@0.5)

While earlier deep learning approaches primarily focused on classification tasks, they often lacked the ability to precisely localize burn boundaries. For instance, although Suha & Sanam [2] achieved a high classification accuracy of 95.6% using VGG16, their method does not provide spatial information critical for surgical planning. Similarly, a recent study by Boissin et al. [26] utilizing a CNN architecture on a comparable dataset reported an accuracy of 87.2%, which highlights the challenges of distinctive feature extraction in burn imagery. In the domain of segmentation-based approaches, which offer greater clinical utility, our proposed model demonstrates a substantial performance leap. Compared to the recent work by Yıldız et al. [27], who achieved a mAP@0.5 of 75.0% using the YOLOv7 architecture, our YOLOv11x-seg model reached a mAP@0.5 of 91.0%. This methodological choice is supported by research in analogous fields, such as the work by Amin et al. [28], who successfully used a modified YOLOv2 architecture to localize diabetic foot ulcers. By leveraging a state-of-the-art YOLOv11-seg architecture, our study provides a more comprehensive diagnostic tool that aligns with the need for both accurate classification and precise spatial assessment in clinical practice.

In conclusion, this study provides robust evidence for the effectiveness of the YOLOv11x-seg architecture in the complex task of burn-wound segmentation and classification, establishing its potential as a valuable clinical decision-support tool. The model’s high accuracy and real-time processing capabilities offer an opportunity to streamline clinical workflows, reduce diagnostic subjectivity, and ultimately improve patient outcomes, particularly in emergency settings where rapid assessment is critical. However, to ensure robust generalizability, future work must focus on validating the model on larger, multi-center datasets that encompass a wider variety of burn types, skin phototypes, and imaging conditions. Furthermore, integrating Explainable AI techniques will be crucial for fostering clinical trust and facilitating adoption by making the model’s decision-making process more transparent. Advancing this technology along these future directions will enable the reliable and widespread integration of AI-assisted tools into critical medical decision-making processes, thereby enhancing the quality and consistency of burn care.

## Conclusions

This study demonstrated the successful application of modern YOLO-based architectures for the automated classification of skin burn depth from digital images. In a comparative analysis, the YOLOv11x-seg model was identified as the superior architecture, achieving an mAP@0.5 of 0.91 and an F1-Score of 0.87. The findings indicate that advanced deep learning models, when trained on a well-curated, multi-source dataset, offer a rapid, objective, and reproducible alternative to traditional subjective evaluation methods. The YOLOv11x-seg model, in particular, shows significant promise for integration into clinical decision support systems, where its ability to accurately segment and classify burn wounds in real-time can enhance triage, accelerate treatment planning, and improve diagnostic consistency. Ultimately, this research provides strong evidence for the effectiveness of next-generation segmentation models in complex medical imaging tasks and establishes a foundation for future work in developing clinically applicable AI tools that can support healthcare professionals in critical decision-making scenarios.

### Impact of hyperparameter optimization and model architectures

Our experimental results demonstrated that the choice of model architecture and systematic hyperparameter tuning significantly influenced the segmentation performance. Specifically, the transition from YOLOv8 to the YOLOv11 architecture provided a structural advantage; the integration of a MobileNetv3-based backbone and C3k2 blocks enhanced the model’s ability to extract features from burn lesions with complex textures and variable boundaries. Furthermore, hyperparameter optimization using evolutionary algorithms—specifically, tuning the initial learning rate (lr0: 0.01) and momentum (0.937)—yielded substantial performance gains. For instance, the YOLOv11x-seg model initially achieved a baseline mAP@0.5 of approximately 0.88, which improved to 0.91 post-optimization. This 3% gain underscores that, although default hyperparameters are robust for general object detection, they are often suboptimal for specific medical imaging tasks that require fine-grained differentiation among burn degrees. These findings suggest that for clinical applications constrained by limited data, maximizing model potential through architectural selection and methodical optimization is as critical as data quality [29,30].

### Limitations and considerations

Despite the promising results and the strength of a multi-source dataset, several limitations must be acknowledged. First, although combining a proprietary dataset with two public repositories from Roboflow Universe and Kaggle enhanced data diversity, inherent biases may still exist within these collections with respect to imaging conditions, patient demographics, or burn etiologies. The model’s generalizability to clinical environments and populations outside of those represented in these three specific datasets has yet to be confirmed through broader, multi-center validation. Second, the confusion matrix analysis revealed that the model occasionally struggled to distinguish between visually similar adjacent burn degrees, a challenge that reflects the intrinsic difficulty of fine-grained burn assessment. Finally, the substantial computational resources required to train a high-capacity model such as YOLOv11x-seg pose challenges for reproducibility and deployment in resource-limited settings, underscoring the need for future research on more lightweight and explainable architectures.

### Recommendations for future studies and research directions

Given the study’s empirical findings and limitations, several promising directions for future research are identified. A primary objective should be to validate the model’s robustness and generalization capability on larger, more diversified datasets compiled from multiple clinical centers. Such datasets would enable a more in-depth understanding of the model’s performance across variables such as skin phototypes, burn etiologies, and inconsistent imaging conditions. To address the challenges of clinical deployment and reproducibility, future studies should also explore lightweight model architectures that can operate efficiently in resource-constrained environments without significantly compromising performance. Finally, recognizing that the “black box” nature of complex models can hinder their adoption, the integration of Explainable AI (XAI) techniques is of great importance. Visualizing the features the model focuses on would enhance transparency, build clinical trust, and provide valuable insights to healthcare professionals, ultimately facilitating the reliable integration of this technology into routine clinical practice.

## References

[1] Liu H, Yue K, Cheng S, Li W, Fu Z. A framework for automatic burn image segmentation and burn depth diagnosis using deep learning. Comput Math Methods Med. 2021;2021:5514224. doi: 10.1155/2021/5514224 33880130 PMC8046560

[2] Suha SA, Sanam TF. A deep convolutional neural network-based approach for detecting burn severity from skin burn images. Mach Learn Appl. 2022;9:100371. doi: 10.1016/j.mlwa.2022.100371

[3] Resch TR, Drake RM, Helmer SD, Jost GD, Osland JS. Estimation of burn depth at burn centers in the United States: a survey. J Burn Care Res. 2014;35(6):491–7. doi: 10.1097/BCR.0000000000000031 25144808

[4] Huang S, Dang J, Sheckter CC, Yenikomshian HA, Gillenwater J. A systematic review of machine learning and automation in burn wound evaluation: a promising but developing frontier. Burns. 2021;47(8):1691–704. doi: 10.1016/j.burns.2021.07.007 34419331

[5] Cirillo MD, Mirdell R, Sjöberg F, Pham TD. Time-independent prediction of burn depth using deep convolutional neural networks. J Burn Care Res. 2019;40(6):857–63. doi: 10.1093/jbcr/irz103 31187119

[6] Wilson RH, Rowland R, Kennedy GT, Campbell C, Joe VC, Chin TL. Review of machine learning for optical imaging of burn wound severity assessment. J Biomed Optics. 2024;29(2):020901. doi: 10.1117/1.JBO.29.2.020901 PMC10869118 38361506

[7] Crouzet C, Nguyen JQ, Ponticorvo A, Bernal NP, Durkin AJ, Choi B. Acute discrimination between superficial-partial and deep-partial thickness burns in a preclinical model with laser speckle imaging. Burns. 2015;41(5):1058–63. doi: 10.1016/j.burns.2014.11.018 25814299 PMC4479308

[8] Laggner M, Lingitz M-T, Copic D, Direder M, Klas K, Bormann D, et al. Severity of thermal burn injury is associated with systemic neutrophil activation. Sci Rep. 2022;12(1):1654. doi: 10.1038/s41598-022-05768-w 35102298 PMC8803945

[9] Jeschke MG, van Baar ME, Choudhry MA, Chung KK, Gibran NS, Logsetty S. Burn injury. Nat Rev Dis Primers. 2020;6(1):11. doi: 10.1038/s41572-020-0145-5 32054846 PMC7224101

[10] Pencle F, Mowery ML, Zulfiqar H. First degree burn. StatPearls. 2023. 28723050

[11] Baker RA, Jones S, Sanders C, Sadinski C, Martin-Duffy K, Berchin H, et al. Degree of burn, location of burn, and length of hospital stay as predictors of psychosocial status and physical functioning. J Burn Care Rehabil. 1996;17(4):327–33. doi: 10.1097/00004630-199607000-00008 8844353

[12] Nielson CB, Duethman NC, Howard JM, Moncure M, Wood JG. Burns: pathophysiology of systemic complications and current management. J Burn Care Res. 2017;38(1):e469–81. doi: 10.1097/BCR.0000000000000355 27183443 PMC5214064

[13] Wearn C, Lee KC, Hardwicke J, Allouni A, Bamford A, Nightingale P, et al. Prospective comparative evaluation study of Laser Doppler Imaging and thermal imaging in the assessment of burn depth. Burns. 2018;44(1):124–33. doi: 10.1016/j.burns.2017.08.004 29032974

[14] Sen CK, Ghatak S, Gnyawali SC, Roy S, Gordillo GM. Cutaneous imaging technologies in acute burn and chronic wound care. Plast Reconstr Surg. 2016;138(3 Suppl):119S–128S. doi: 10.1097/PRS.0000000000002654 27556752 PMC5207795

[15] Ganapathy P, Tamminedi T, Qin Y, Nanney L, Cardwell N, Pollins A, et al. Dual-imaging system for burn depth diagnosis. Burns. 2014;40(1):67–81. doi: 10.1016/j.burns.2013.05.004 23790396

[16] Burmeister DM, Cerna C, Becerra SC, Sloan M, Wilmink G, Christy RJ. Noninvasive techniques for the determination of burn severity in real time. J Burn Care Res. 2017;38(1):e180–91. doi: 10.1097/BCR.0000000000000338 27355653

[17] Miller DD, Brown EW. Artificial intelligence in medical practice: the question to the answer? Am J Med. 2018;131(2):129–33. doi: 10.1016/j.amjmed.2017.10.035 29126825

[18] Shen D, Wu G, Suk H-I. Deep learning in medical image analysis. Annu Rev Biomed Eng. 2017;19:221–48. doi: 10.1146/annurev-bioeng-071516-044442 28301734 PMC5479722

[19] Jung K-H, Park H, Hwang W. Deep learning for medical image analysis: applications to computed tomography and magnetic resonance imaging. Hanyang Med Rev. 2017;37(2):61. doi: 10.7599/hmr.2017.37.2.61

[20] Khanam R, Hussain M. YOLOv11: An Overview of the Key Architectural Enhancements. 2024 [cited 14 May 2025]. Available from: https://arxiv.org/abs/2410.17725v1

[21] Redmon J, Divvala S, Girshick R, Farhadi A. You only look once: Unified, real-time object detection. Proceedings of the IEEE Computer Society Conference on Computer Vision and Pattern Recognition. 2016. pp. 779–788. doi: 10.1109/CVPR.2016.91

[22] Ragab MG, Abdulkadir SJ, Muneer A, Alqushaibi A, Sumiea EH, Qureshi R, et al. A comprehensive systematic review of YOLO for medical object detection (2018 to 2023). IEEE Access. 2024;12:57815–36. doi: 10.1109/access.2024.3386826

[23] Wang CY, Liao HYM. YOLOv1 to YOLOv10: The fastest and most accurate real-time object detection systems. 2024. [cited 14 May 2025]. 10.1561/116.20240058

[24] Zafar I, Tzanidou G, Burton R, Patel N, Araujo L. Hands-On Convolutional Neural Networks with TensorFlow: Solve Computer Vision Problems with Modeling in TensorFlow and Python. 2018.

[25] Karthik J, Nath GS, Veena A. Deep Learning-based approach for skin burn detection with multi-level classification. Lecture Notes in Electrical Engineering. Springer Singapore; 2021. pp. 31–40. doi: 10.1007/978-981-33-6987-0_3

[26] Boissin C, Laflamme L, Fransén J, Lundin M, Huss F, Wallis L, et al. Development and evaluation of deep learning algorithms for assessment of acute burns and the need for surgery. Sci Rep. 2023;13(1):1794. doi: 10.1038/s41598-023-28164-4 36720894 PMC9889389

[27] Yıldız M, Sarpdağı Y, Okuyar M, Yildiz M, Çiftci N, Elkoca A, et al. Segmentation and classification of skin burn images with artificial intelligence: Development of a mobile application. Burns. 2024;50(4):966–79. doi: 10.1016/j.burns.2024.01.007 38331663

[28] Amin J, Sharif M, Anjum MA, Khan HU, Malik MSA, Kadry S. An integrated design for classification and localization of diabetic foot ulcer based on CNN and YOLOv2-DFU models. IEEE Access. 2020;8:228586–97. doi: 10.1109/access.2020.3045732

[29] Andonie R. Hyperparameter optimization in learning systems. J Membr Comput. 2019;1(4):279–91. doi: 10.1007/s41965-019-00023-0

[30] He L, Zhou Y, Liu L, Ma J. Research and application of YOLOv11-based object segmentation in intelligent recognition at construction sites. Buildings. 2024;14(12):3777. doi: 10.3390/buildings14123777

